# TRPV4 contributes to ER stress and inflammation: implications for Parkinson’s disease

**DOI:** 10.1186/s12974-022-02382-5

**Published:** 2022-01-29

**Authors:** Na Liu, Liping Bai, Zhipeng Lu, Rou Gu, Dongdong Zhao, Fang Yan, Jie Bai

**Affiliations:** 1 Medical School, Kunming University of Science and Technology, No.727 Jingming South Road, Kunming, 650500 China; 2 Department of Anesthesiology, The First People’s Hospital of Yunnan Province, The Affiliated Hospital of Kunming University of Science and Technology, Kunming, 650032 China

**Keywords:** TRPV4, MPTP, Parkinson’s disease, SN, ER stress, Inflammation

## Abstract

**Background:**

Parkinson’s disease (PD) is a progressive neurodegenerative disorder. Its molecular mechanism is still unclear, and pharmacological treatments are unsatisfactory. Transient receptor potential vanilloid 4 (TRPV4) is a nonselective Ca^2+^ channel. It has recently emerged as a critical risk factor in the pathophysiology of neuronal injuries and cerebral diseases. Our previous study reported that TRPV4 contributed to endoplasmic reticulum (ER) stress in the MPP^+^-induced cell model of PD. In the present study, we detected the role and the mechanism of TRPV4 in 1-Methyl-4-phenyl-1,2,3,6-tetrahydropyridine (MPTP)-induced PD mice.

**Methods:**

Intracerebral injection of an adeno-associated virus (AAV) into the substantia nigra (SN) of mice was used to knockdown or upregulate the expression of TRPV4 and intraperitoneal injection of MPTP. Rotarod and pole tests were used to evaluate the locomotor ability of mice. We used immunohistochemistry, Nissl staining and Western blot to detect the alterations in the number of tyrosine hydroxylase (TH)-positive neurons, Nissl-positive neurons, the levels of ER stress-associated molecules and proinflammatory cytokines in the SN.

**Results:**

The SN was transfected with AAV for 3 weeks and expressed the target protein with green fluorescence. Knockdown of TRPV4 via injection of a constructed AAV-TRPV4 shRNAi into the SN alleviated the movement deficits of PD mice. Upregulation of TRPV4 via injection of a constructed AAV-TRPV4 aggravated the above movement disorders. The expression of TRPV4 was upregulated in the SN of MPTP-treated mice. Injection of AAV-TRPV4 shRNAi into the SN rescued the number of TH-positive and Nissl-positive neurons in the SN decreased by MPTP, while injection of AAV-TRPV4 induced the opposite effect. Moreover, MPTP-decreased Sarco/endoplasmic reticulum Ca^2+^-ATPase 2 (SERCA2) and pro-cysteinyl aspartate specific proteinase-12 (procaspase-12), MPTP-increased Glucose-regulated protein 78 (GRP78), Glucose-regulated protein 94 (GRP94) and C/EBP homologous protein (CHOP) were inhibited by AAV-TRPV4 shRNAi infection, and enhanced by AAV-TRPV4. In the same way, MPTP-decreased procaspase-1, MPTP-increased Interleukin-18 (IL-18), Cyclooxgenase-2 (COX-2) and 5-Lipoxygenase (5-LOX) were inhibited by AAV-TRPV4 shRNAi, or further exacerbated by AAV-TRPV4.

**Conclusions:**

These results suggest that TRPV4 mediates ER stress and inflammation pathways, contributing to the loss of dopamine (DA) neurons in the SN and movement deficits in PD mice. Moreover, this study provides a new perspective on molecular targets and gene therapies for the treatment of PD in the future.

## Background

Parkinson’s disease (PD) is a common neurodegenerative disease characterized by the loss of dopamine (DA) neurons in the substantia nigra (SN) and a decrease in DA content in the striatum. The pathogenesis of PD is related to environmental factors. 1-Methyl-4-phenyl-1,2,3,6-tetrahydropyridine (MPTP), an environmental neurotoxin, is widely used to produce the animal models for the study of PD, especially the rodent models [1, 2]. Although the mechanism of PD remains elusive, increasing evidence indicates that ER stress is involved [3, 4]. The endoplasmic reticulum (ER) plays a crucial role in maintaining cellular function and Ca^2+^ homeostasis. When ER stress occurs, the Ca^2+^-induced excitotoxicity or Ca^2+^ overload is considered as an essential mechanism linked to neuronal apoptosis. Our previous study showed that upregulation of TRPV4 induced by the 1-methyl-4-phenylpyridinium ion (MPP^+^) mediated ER stress and contributed to PC12 cell apoptosis [5]. The role of TRPV4 in regulating neurotoxicity in vitro was first reported in the study. It was also identified that HC067047 (a special inhibitor of TRPV4) had an antiapoptotic effect. This supports the view that disorder of Ca^2+^ homeostasis is a primary feature of the pathogenesis of PD but not the result of the neurodegenerative process. In addition, neuroinflammation refers to the inflammatory process occurring in nervous system. Clinical data indicate higher levels of the proinflammatory cytokines interleukin-6 (IL-6) and interleukin-1β (IL-1β) and tumor necrosis factor-α (TNF-α) in the serum and cerebrospinal fluid of PD patients [6, 7]. Neuroinflammation has been linked to PD and is responsible for DA neuron death in the SN. In addition to astrocytes and microglia, neurons also release cytokines and chemokines, and induce a robust inflammatory response [8]. Correspondingly, one study has shown that the dysfunction of Ca^2+^ homeostasis is responsible for the high levels of inflammatory factors leading to epileptic activity [9]. Moreover, inhibiting transient receptor potential (TRP) channel may decrease the influx of Ca^2+^ and regulate morphine-induced neurodegeneration of neurons in the hippocampus by decreasing inflammation [10].

Transient receptor potential vanilloid 4 (TRPV4), a member of the TRP family, is a nonselective Ca^2+^ channel involved in diverse pathological and physiological responses. It is more permeable to Ca^2+^ than other cations [11]. It is widely expressed in neurons in the brain [12, 13]. When it is activated by endogenous and exogenous stimuli, the influx of Ca^2+^ is increased and results in overloaded of intracellular free Ca^2+^ [14, 15]. Therefore, hyperactivation of TRPV4 leads to cell apoptosis in many pathological processes [16–18] and acts as a risk factor for the development of brain diseases [19], including cerebral ischemic reperfusion injury [20, 21], intracerebral hemorrhage [22], brain edema [23, 24], epilepsy [25], and Alzheimer’s disease [26, 27]. In contrast, the TRPV4 antagonist HC067047 exerts a neuroprotective effect by inhibiting the unfolded protein response (UPR) and the inflammation signaling pathways [22, 25]. Thus, the contribution of TRPV4 on cytotoxicity has received more attention from researchers. However, to the best of our knowledge, the role of TRPV4 in regulating neurotoxicity in the MPTP-induced mouse model is unknown.

Neurodegenerative diseases such as PD impose a heavy burden on the aging society. The currently available treatment approaches for PD are limited. PD is normally controlled by drugs. However, as PD continues to advance, presently available pharmacotherapies often fail to effectively relieve symptoms and sometimes cause side effects [28, 29]. The new treatment strategies for PD are urgently needed.

Based on a previous study in vitro, we stereotaxically injected AAV2/9 bilaterally into the SN of mice to regulate TRPV4 gene expression and then intraperitoneally injected MPTP. We examined the expression of TRPV4 and assessed its contribution to ER stress and inflammation induced by MPTP in vivo. These results improve the understanding on the pathogenesis of PD and provide a novel therapeutic target with PD.

## Methods

### Animals

Male C57BL/6J mice (20–25 g, 7–8 weeks) were obtained from Chongqing Medical University (Chongqing, China). Mice were housed individually in a plastic cage under a 12 h light/dark cycle (lights on at 8:00 a.m.) with ad libitum access to water and food. The housing room temperature was maintained at 20–26 °C, and the humidity was 40–70%. The animals were acclimatized to this laboratory environment for at least 1 week prior to experimental procedures. The experimenter was blind to the treatment groups at the time of the tests and analyses. Surgeries and behavioral assays were carried out at the Laboratory Animal Center Facilities of Kunming University of Science and Technology.

### Drug treatment and viral vectors

MPTP was purchased from Sigma-Aldrich (# M0896). AAV2/9 TRPV4 shRNA (1.5 × 10^12^ vg/ml, AAV-TRPV4 shRNAi) was constructed to knockdown TRPV4, and AAV2/9 TRPV4 (1.6 × 10^12^ vg/ml, AAV-TRPV4) was used to upregulate TRPV4 expression, and were purchased from Hanbio Biotechnology Co. (Shanghai, China).

### Animal model

For AAV2/9 injection, mice were anesthetized with pentobarbital sodium salt (50 mg/kg) through intraperitoneal injections and placed in a stereotaxic frame (RWD Life Science Co, Ltd, China). The mice were infused with 0.5 μl AAV-TRPV4 shRNAi or 0.3 μl AAV-TRPV4 per side into the SN (3.0 mm posterior to bregma; 1.0 mm lateral to the midline; 4.5 mm below the dural surface) [30] with using an electric microinjection pump (World Precision Instruments Inc. Shanghai, China) at a rate of 250 nl/min, and the needle was left in place for an additional 5 min period before it was slowly retracted. After 21 days, we randomly selected one mouse from each AAV-injected group to obtain frozen sections, and confirmed the accuracy of transfection site by green fluorescent protein (GFP) under a fluorescence microscope (Leica, Germany). Based on the previous study, the left mice were administered with MPTP (30 mg/kg) and saline by intraperitoneal injections for 7 days at an interval of 24 h [31].

### Fluorescence imaging

The mice were sacrificed by cervical vertebra dislocation. To obtain frozen sections, the brains were fixed in 4% paraformaldehyde (PFA) solution overnight at 4 °C and subsequently incubated in 30% sucrose to dehydrate overnight at 4 °C. Then, the brains were frozen in the freezing microtome (Leica, Germany), and the samples were cut into coronal slices at 25 μm thickness in the region − 3.00 mm from the bregma for examination GFP under a fluorescence microscope (Leica, Germany).

### Movement behavioral tests

#### Pole test

A vertical wooden pole with a rough surface (5 cm in height and 1 cm in diameter) was placed in the home cage. The mice were placed head-up on top of the pole, oriented themselves downward and descended the length of the pole back into their home cages. The mice received 2 days of training consisting of three trials for each session day. On the test day, the mice were subjected to three trials, and the time spent orienting themselves downward (*T*-turn time) and the time spent descending (*T*-descend time) were measured. The best performance over the three trials was used. If the mouse was unable to turn completely downwards, fall or slip down, the default value of 120 s was recorded, which represented the maximal severity of impairment [32, 33].

#### Rotarod test

Motor performance was tested using an accelerating rotarod (Ugo Basile, Italy). Prior to the test, the mice were trained for 2 days. On the training day, the mice were placed on the rod rotating at a speed of 5–25 rpm for three adaptation sessions, and the duration did not exceed 180 s. At least 5 min of rest was allowed between each trial to alleviate mouse fatigue and stress. On the test day, the mice were subjected to three trials in which the rod accelerated (5–30 rpm). The mice were placed on the rod, and the time it took each mouse to fall from the rod was recorded. The average time was calculated for each mouse and then used for the statistical purpose.

#### Immunohistochemistry

The mice were anesthetized with pentobarbital sodium salt (50 mg/kg) by intraperitoneal injections and slowly perfused through the left ventricle with 0.9% saline at room temperature, followed by ice-cold 4% PFA solution. The brains were rapidly removed and fixed in 4% PFA solution overnight at 4 °C. After fixation, brains were embedded in paraffin following standard procedures and serially sectioned at 6 μm (Leica, Germany) for staining. Coronal sections corresponding to the region − 2.96 to − 3.06 mm from the bregma were selected for analysis. Every 7th and 9th section was used for immunohistochemistry and Nissl staining, respectively. In immunohistochemistry assay, brain sections were deparaffinized with xylene and rehydrated through a serious of ethanol solutions (from 100 to 75%). Typically, the slices were immersed in xylene for 12 min (twice), 100% ethanol for 5 min, 95% ethanol for 5 min, 85% ethanol for 5 min, and 70% ethanol for 5 min and then washed with ultrapure water for 5 min three times. Antigen retrieval was performed by soaking the sections in boiling citrate buffer (pH 6.0) in a microwave for 15 min. Endogenous peroxidase activity was blocked with 3% hydrogen peroxide (H_2_O_2_) for 30 min. The sections were permeabilized with 0.25% Triton-X-100 in phosphate-buffered saline (PBS) for 10 min, followed by incubation with 5% normal goat serum (Thermo Fisher, USA) for 1 h at room temperature. Next, the brain sections were probed with anti-Tyrosine hydroxylase (TH) (1:300; ab137869, Abcam) primary antibody solution at 4 °C overnight. Then, the sections were incubated with secondary antibody (goat anti-rabbit IgG, 1:200; #5450-0010, KPL, USA) at room temperature for 1 h. After being incubated with 3,3′-diaminobenzidine (DAB) (Thermo Fisher, USA) for 10 min, the sections were counterstained with hematoxylin. Typically, the sections were rinsed three times in PBS for 5 min between each incubation period. Images were taken with using a Leica DM1000 microscope (Leica, China).

#### Nissl staining

The slices were dewaxed according to the method mentioned above. Next, the slices were immersed in 2% thionine for 3 min, ultrapure water for 10 s (twice), 100% ethanol for 10 s, 95% ethanol for 10 s, 85% ethanol for 10 s, 70% ethanol for 10 s, xylene for 2 min, and 100% ethanol for 10 s and then washed with ultrapure water for 5 min. Finally, the slices were sealed with neutral gum after desiccation.

#### Stereology and neuron counting

An unbiased stereological estimation of the total number of TH-positive neurons and Nissl-positive neurons in the SN was performed using the optical fractionator method and calculated with Stereo Investigator software (MBF Bioscience). Counting was performed using microscope (10× objective lens). The counted area covered from the rostral tip of substantia nigra pars compacta to the caudal end of substantia nigra pars reticulate. The counting frame was randomly placed on the first counting field and gradually moved through all counting areas. The total number of neurons was estimated according to the optical fractionator formula.

### Western blot analysis

The mice were sacrificed by cervical vertebra dislocation after behavioral tests.

The SN was rapidly dissected out, frozen, and stored in a deep freezer at − 80 °C until analysis. Samples were lysed in the fresh radio immunoprecipitation assay (RIPA) protein lysis buffer. The protein concentration was determined using Bio-Rad protein assay reagent (Hercules, CA, USA). Proteins were separated by sodium dodecyl sulfate–polyacrylamide gel electrophoresis (SDS–PAGE) and transferred onto polyvinylidene fluoride (PVDF) membranes (Millipore Corporation, Billerica, MA, USA). Then, the membranes were soaked in a solution of 10% skim milk or 3% bovine albumin V (in PBS, pH 7.2, containing 0.1% Tween-20) overnight at 4 °C. The membranes were incubated by primary antibodies against TRPV4 (1:1000; ab39260, Abcam), sarco/endoplasmic reticulum Ca^2+^-ATPase 2 (SERCA2, 1:2000; ab137020, Abcam), glucose-regulated protein 78 (GRP78, 1:1000; #11587-1-AP, Proteintech Group), glucose-regulated protein 94 (GRP94, 1:1000; #10979-1-AP, Proteintech Group), pro-cysteinyl aspartate specific proteinase-12 (procaspase-12, 1:1000; ab8118, Abcam), C/EBP homologous protein (CHOP, 1:1000; #15204-1-AP, Proteintech Group), TH (1:1500; ab137869, Abcam), pro-cysteinyl aspartate specific proteinase-1 (procaspase-1, 1:1500; ab238972, Abcam), Cyclooxgenase-2 (COX-2, 1:1000; #66351-1-lg, Proteintech Group), 5-lipoxygenase (5-LOX, 1:1000; ab169755, Abcam), Interleukin-18 (IL-18, 1:1000; ab207323, Abcam) and β-actin (1:10,000; ab115777, Abcam) overnight at 4 °C. After extensive washing, the membranes were incubated with peroxidase conjugated goat anti-mouse or anti-rabbit IgG (#5450-0011, #5450-0010 KPL, USA). The epitopes were visualized with an enhanced chemiluminescence (ECL) Western blot detection kit (Millipore Corporation, USA). Other steps were performed following the instructions of each antibody. Densitometry analysis was performed using ImageJ software.

### Statistical analysis

Statistical analyses were performed by Prism 5 (GraphPad Software). Before data analysis, both normality of the distribution and homogeneity of variance were assessed. All data are presented as mean ± SEM. For assessment of change of a group by a certain intervention, data were analyzed by the two-tailed Student’s *t* test for comparison differences between the control group and the treated group. Data were analyzed by one-way variance (ANOVA) with Bonferroni post hoc test for comparison between multiple groups. *P* value less than 0.05 was considered statistically significant. The significance level is represented as asterisks (*P < 0.05, **P < 0.01, ***P < 0.001).

## Results

### TRPV4 expression was upregulated in MPTP-induced PD mice

**Fig. 1:**
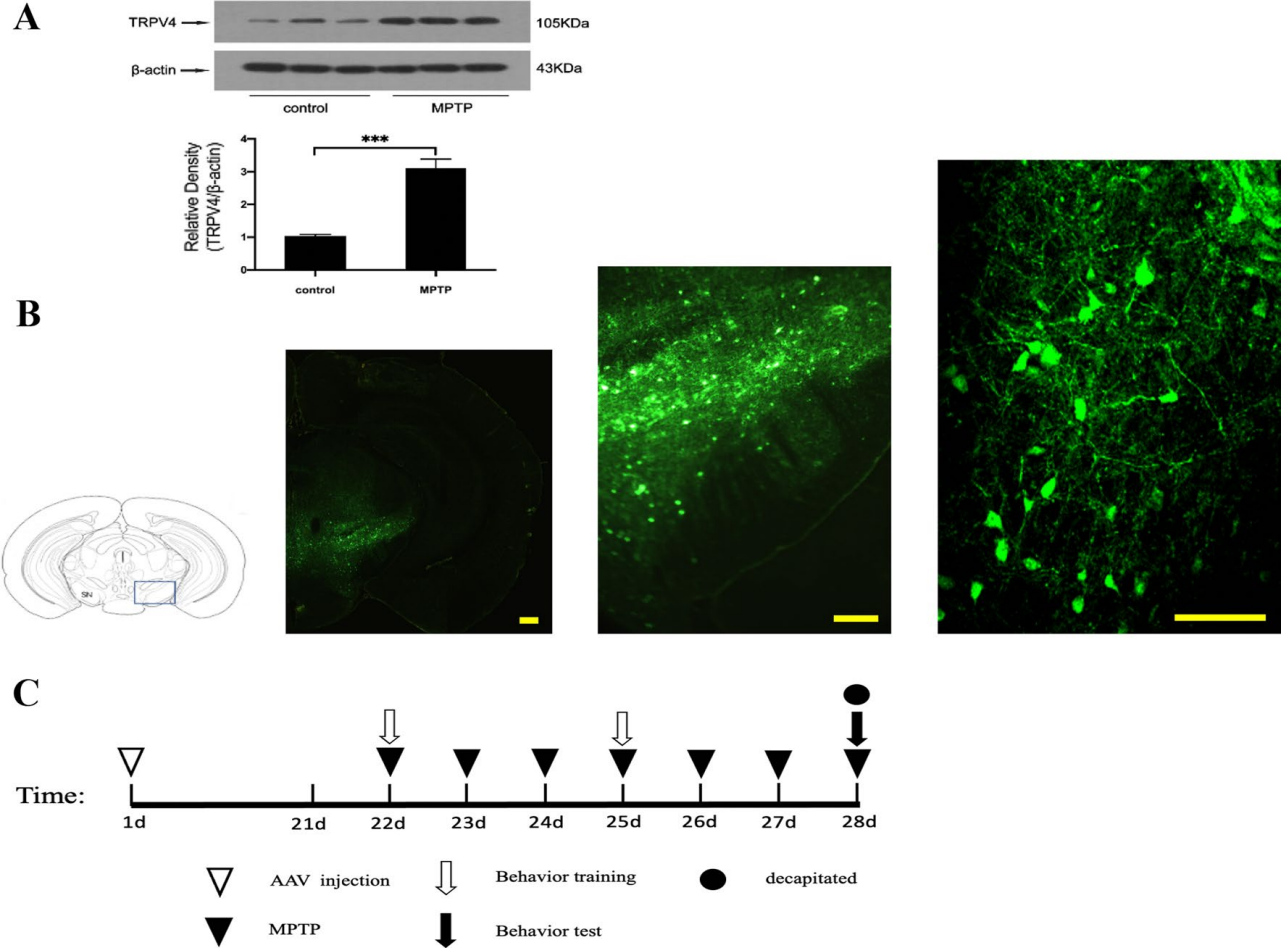
Expression of TRPV4 in the SN after treatment with MPTP and AAV injection. **A** MPTP was administrated intraperitoneally at 30 mg/kg/day for 7 days, mice were sacrificed after the behavioral tests by cervical vertebra dislocation, the SN was rapidly dissected out. The expression of TRPV4 in the SN was assessed by Western blot (*n* = 10). Compared to that in the control group, TRPV4 was upregulated by MPTP. Data are presented as mean ± SEM. Statistical significance: ***P < 0.001. **B** AAV 0.5 μl was stereotaxically injected bilaterally into the SN at a rate of 250 nl/min, 21 days later the brains were dissected and cut into coronal slices at 25 μm thickness, fluorescence images of coronal frozen sections at different magnifications were presented by GFP in neurons in the SN region. Scale bar = 100 μm. **C** Schematic illustration of the experimental schedule

Based on our previous study in vitro, we first detected the expression of TRPV4 in the SN of MPTP-induced mice. We found that the level of TRPV4 was significantly increased compared with that in vehicle-treated control mice (Fig. 1A). This result suggests that TRPV4 may play an essential role in MPTP-induced neurotoxicity. To further confirm this hypothesis, we next infused AAV-GFP-packaged mouse shRNAi or the full-length TRPV4 gene by intracerebral injection to knockdown or upregulate the expression of TRPV4 in the SN, respectively. By 3 weeks post AAV injection, the SN was transfected and expressed the target protein with green fluorescence (Fig. 1B). Subsequently, we administered MPTP intraperitoneally following the schedule below (Fig. 1C).

### The effect of TRPV4 on the behavioral performance of PD mice

**Fig. 2:**
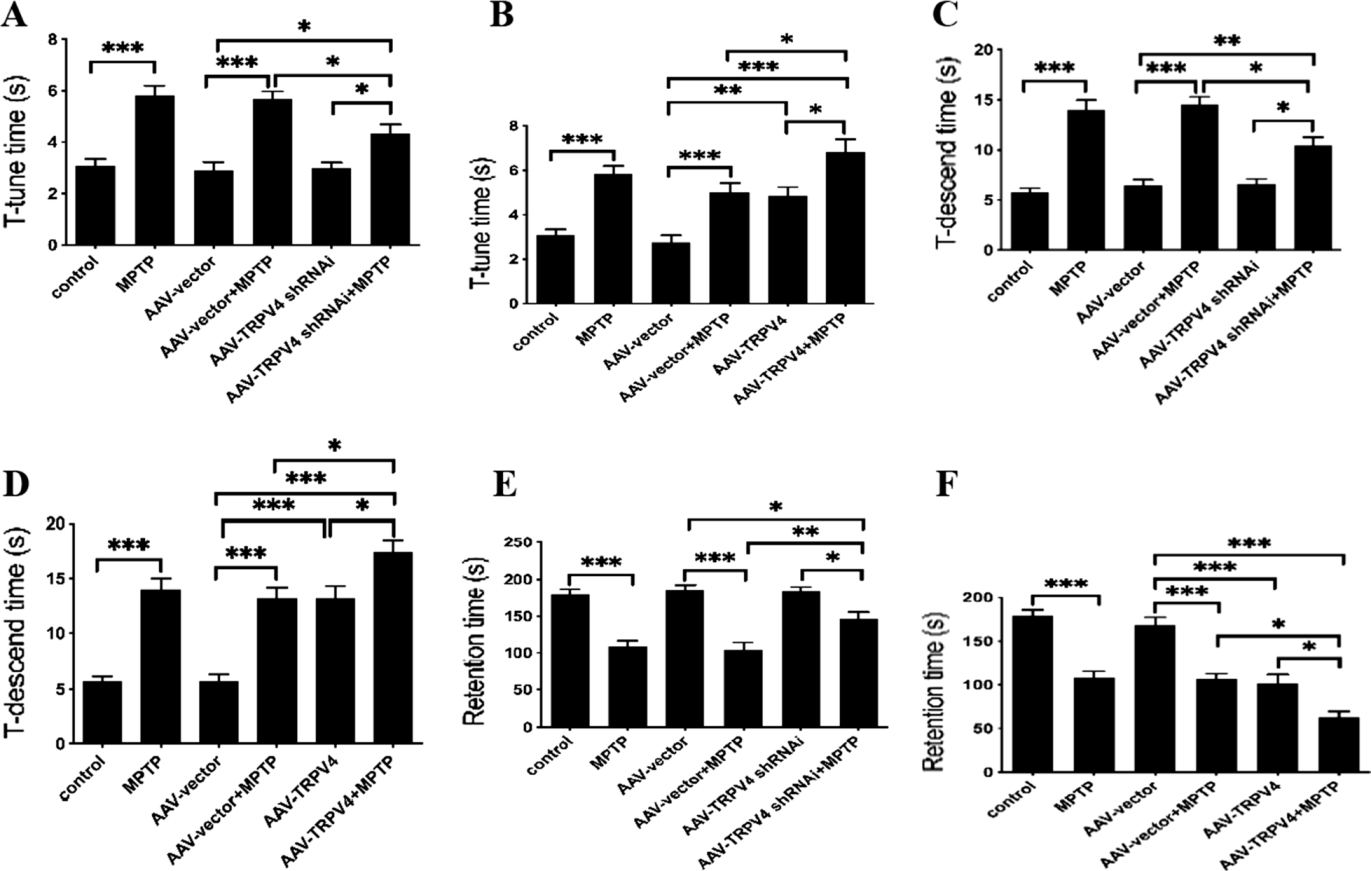
TRPV4 affected the behavioral performances of PD mice. After infused with 0.5 μl AAV-TRPV4 shRNAi or 0.3 μl AAV-TRPV4 bilaterally into the SN for 21 days and administrated with MPTP at 30 mg/kg/day for 7 days, mice were subjected to movement behavioral tests, including the pole test and rotarod test. **A** Knockdown of TRPV4 restored the *T*-tune time of PD mice (*n* = 12). **B** Upregulation of TRPV4 further prolonged the *T*-tune time of PD mice (*n* = 12). **C** Knockdown of TRPV4 restored the *T*-descend time of PD mice (*n* = 12). **D** Upregulation of TRPV4 further prolonged the *T*-descend time of PD mice (*n* = 12). **E** Knockdown of TRPV4 restored the retention time of PD mice (*n* = 12). **F** Upregulation of TRPV4 further shortened the retention time of PD mice (*n* = 12). For each condition, data were collected from three trials for each mouse. All data are presented as mean ± SEM. Significant differences: *P < 0.05, **P < 0.01, ***P < 0.001

The pole and rotarod tests are used to assess the agility and bradykinesia in PD mice and could sensitively detect nigrostriatal dysfunction [33]. The results of the tests indicated that behavioral performances were significantly different among the groups. Post hoc analysis revealed that motor disorders were induced in the group exposed to MPTP for 7 days compared with the control group. Knockdown of TRPV4 alleviated the movement deficits of PD mice. AAV-TRPV4 shRNAi restored the *T*-tune time, the *T*-descend time and the retention time of PD mice (Fig. 2A, C, E). Upregulation of TRPV4 further prolonged the *T*-tune time and the *T*-descend time, and greatly shortened the retention time of PD mice (Fig. 2B, D, F). These motor alterations indicated a pivotal role of TRPV4 in movement deficits in PD mice.

### TRPV4 expression was significantly correlated with TH-positive neuron survival in the SN

**Fig. 3:**
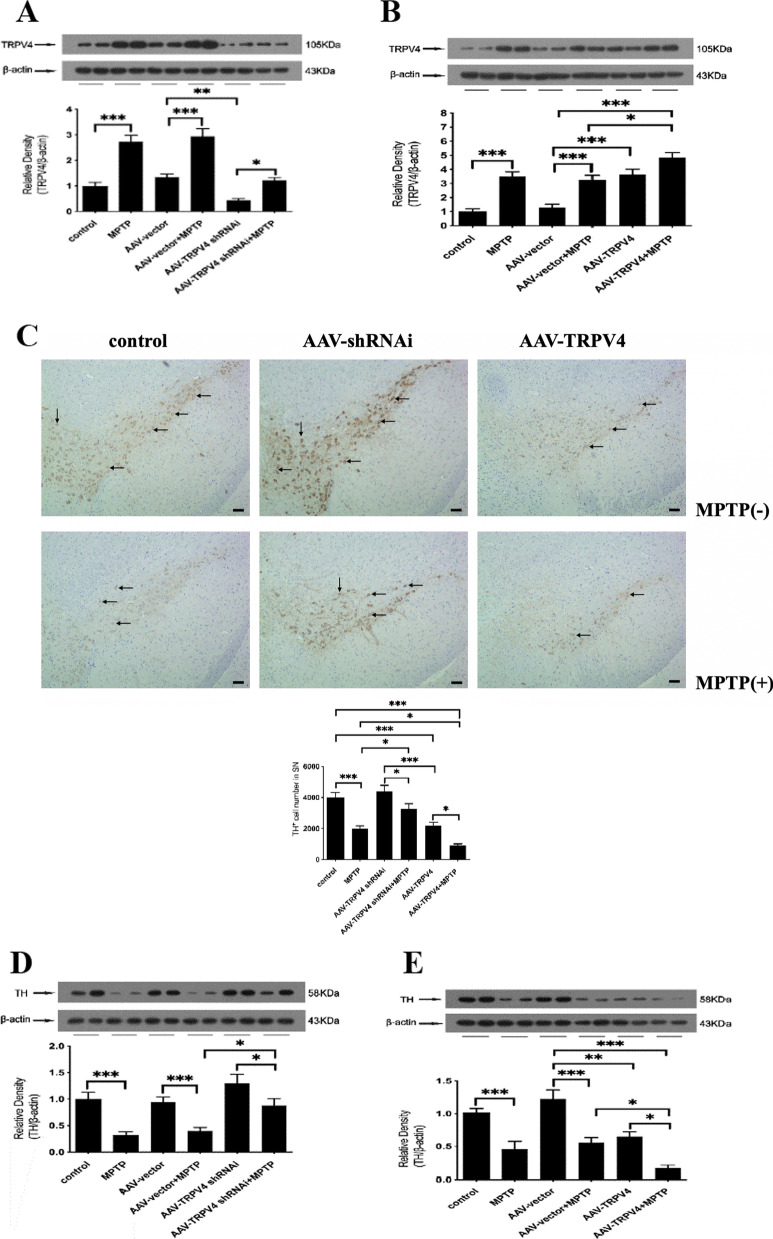
TRPV4 affected the number of TH-positive neurons in the SN. After infused with 0.5 μl AAV-TRPV4 shRNAi or 0.3 μl AAV-TRPV4 bilaterally into the SN of mice for 21 days and administrated with MPTP at 30 mg/kg/day for 7 days, the SN was rapidly dissected out. The expressions of TRPV4 and TH in the SN were detected by Western blot. **A** AAV-TRPV4 shRNAi downregulated the expression of TRPV4 in the SN, and significantly inhibited the MPTP-induced increase in TRPV4 (*n* = 6). **B** AAV-TRPV4 upregulated the expression of TRPV4 in the SN, and further increased the MPTP-induced high levels of TRPV4 (*n* = 6). **C** Representative sample of brain slices with immunohistochemistry staining of TH-positive neurons in the SN of mice. Knockdown of TRPV4 rescued the number of TH-positive neurons, while upregulation of TRPV4 exacerbated the already reduction in the number of TH-positive neurons in the SN of PD mice (*n* = 3). **D** Knockdown of TRPV4 restored the expression of TH in the SN of PD mice (*n* = 6). **E** Upregulation of TRPV4 further decreased the expression of TH in the SN of PD mice (*n* = 6). All data are presented as mean ± SEM. Significant differences: *P < 0.05, **P < 0.01, ***P < 0.001

To explore the role of TRPV4 in PD mice, we regulated the expression of TRPV4 in the SN by AAV transfection. By Western blot, we found that TRPV4 expression was changed mainly in the SN in each group (Fig. 3A, B). Furthermore, knockdown of TRPV4 rescued the number of TH-positive neurons in the SN, while upregulation of TRPV4 exacerbated the reduction in the number of TH-positive neurons in PD mice (Fig. 3C). These results were confirmed by detecting the expression of TH by Western blot (Fig. 3D, E).

### TRPV4 expression was significantly correlated with neurodegeneration in the SN

**Fig. 4:**
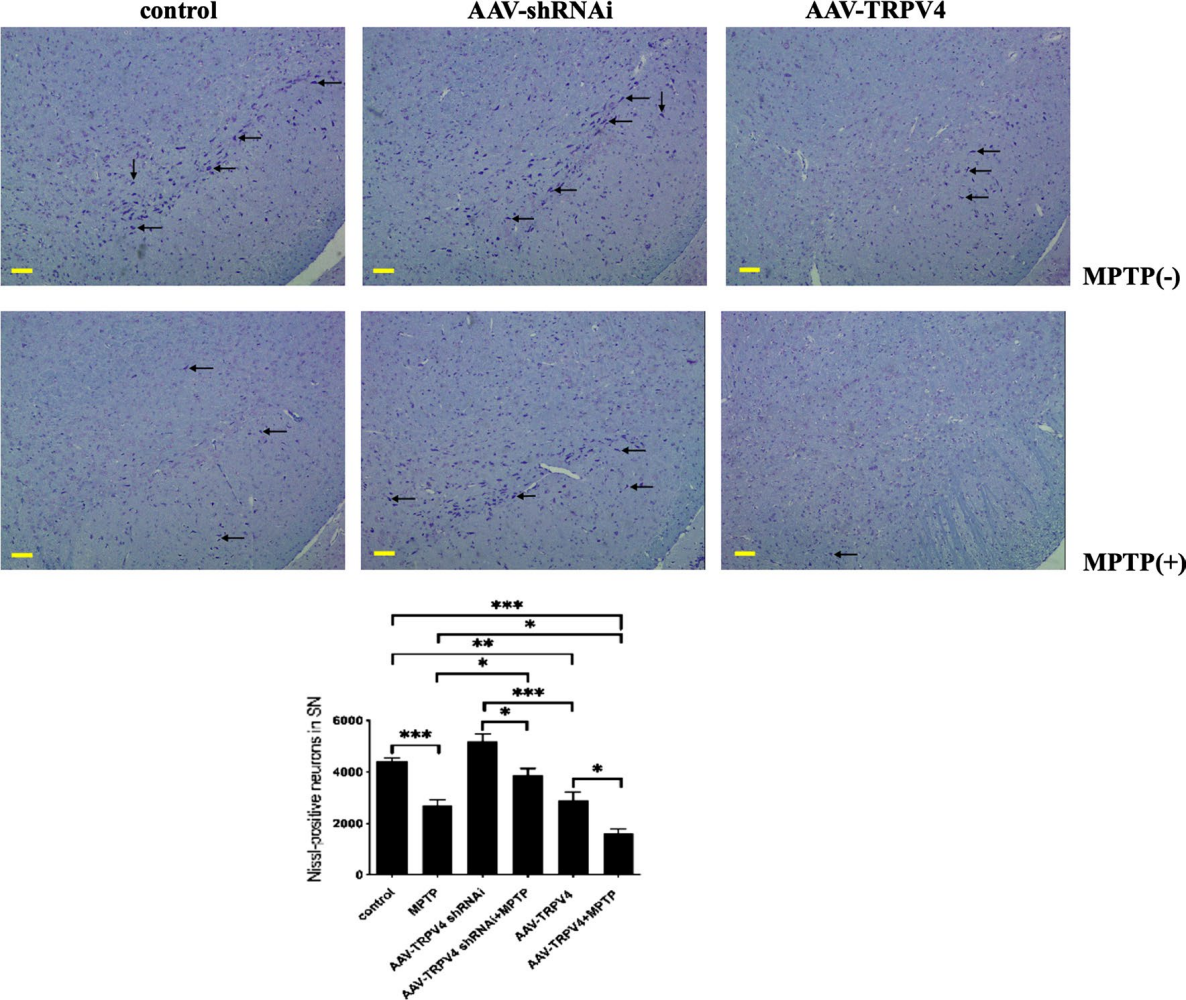
TRPV4 affected the number of Nissl-positive neurons in the SN. After infused with 0.5 μl AAV-TRPV4 shRNAi or 0.3 μl AAV-TRPV4 bilaterally into the SN of mice for 21 days and administrated with MPTP at 30 mg/kg/day for 7 days, representative sample of brain slices with Nissl staining showed Nissl-positive neurons in the SN. Knockdown of TRPV4 rescued the number of Nissl-positive neurons in the SN of PD mice, while upregulation of TRPV4 exacerbated the already reduction in the number of Nissl-positive neurons in the SN of PD mice (bar: 100 μm, *n* = 3). Data are presented as mean ± SEM. Significant differences: *P < 0.05, **P < 0.01, ***P < 0.001

To further determine the role of TRPV4 in PD mice, we performed Nissl staining in the SN. We found that knockdown of TRPV4 rescued the number of Nissl-positive neurons, while upregulation of TRPV4 exacerbated the decrease in the number of Nissl-positive neurons in the SN of PD mice (Fig. 4). Stereological analysis showed that TRPV4 played a crucial role in neurodegeneration.

### Knockdown of TRPV4 in the SN attenuated ER stress induced by MPTP

**Fig. 5:**
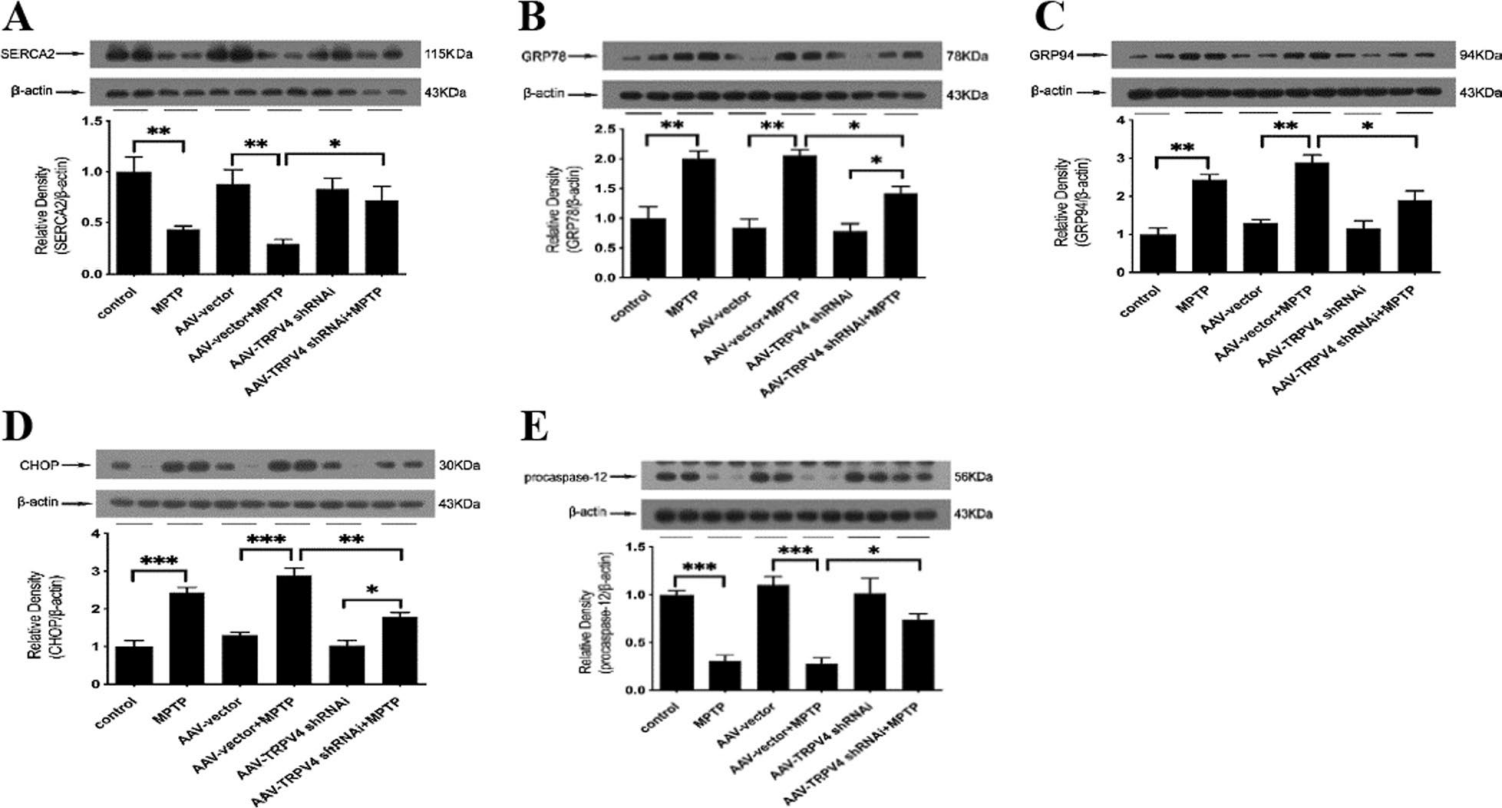
TRPV4 knockdown attenuated MPTP-induced ER stress in the SN. After infused with 0.5 μl AAV-TRPV4 shRNAi bilaterally into the SN of mice for 21 days and administrated with MPTP at 30 mg/kg/day for 7 days, the SN was rapidly dissected out. The protein levels of SERCA2, GRP78, GRP94, CHOP and procaspase-12 in the SN of mice were detected by Western blot. **A** Knockdown of TRPV4 restored the MPTP-induced decrease in SERCA2 (*n* = 6). **B** Knockdown of TRPV4 inhibited the MPTP-induced upregulation of GRP78 (*n* = 6). **C** Knockdown of TRPV4 inhibited the MPTP-induced upregulation of GRP94 (*n* = 6). **D** Knockdown of TRPV4 inhibited the MPTP-induced upregulation of CHOP (*n* = 6). **E** Knockdown of TRPV4 partly inhibited the MPTP-induced decrease in procaspase-12 (*n* = 6). All data are presented as mean ± SEM. Statistical significance: *P < 0.05, **P < 0.01, ***P < 0.001

Based on the findings presented above, we then investigated whether TRPV4 in the SN could serve as a key determinant of ER stress and thus induce the loss of neurons. To examine this possibility, we constructed an AAV2/9 TRPV4 shRNAi, which was infused bilaterally into the SN for 21 days to knockdown TRPV4 expression. We found that the expression of SERCA2, which is located on the ER and associated with ER stress, was decreased by MPTP and restored by knockdown of TRPV4 (Fig. 5A). Moreover, MPTP-induced activation of the ER-resident chaperones GRP78 and GRP94 was inhibited by AAV-TRPV4 shRNAi infection (Fig. 5B, C), suggesting that knockdown of TRPV4 attenuated MPTP-induced ER stress. To further validate this mechanism of action of TRPV4, we assessed the expression of CHOP and procaspase-12, which are key apoptotic factors associated with ER stress. Knockdown of TRPV4 decreased the expression of CHOP and increased procaspase-12 expression in PD mice (Fig. 5D, E). Taken together, these data indicate that knockdown of TRPV4 exerts its neuroprotective effect by attenuating MPTP-induced ER stress.

### Upregulation of TRPV4 in the SN further aggravated ER stress induced by MPTP

**Fig. 6:**
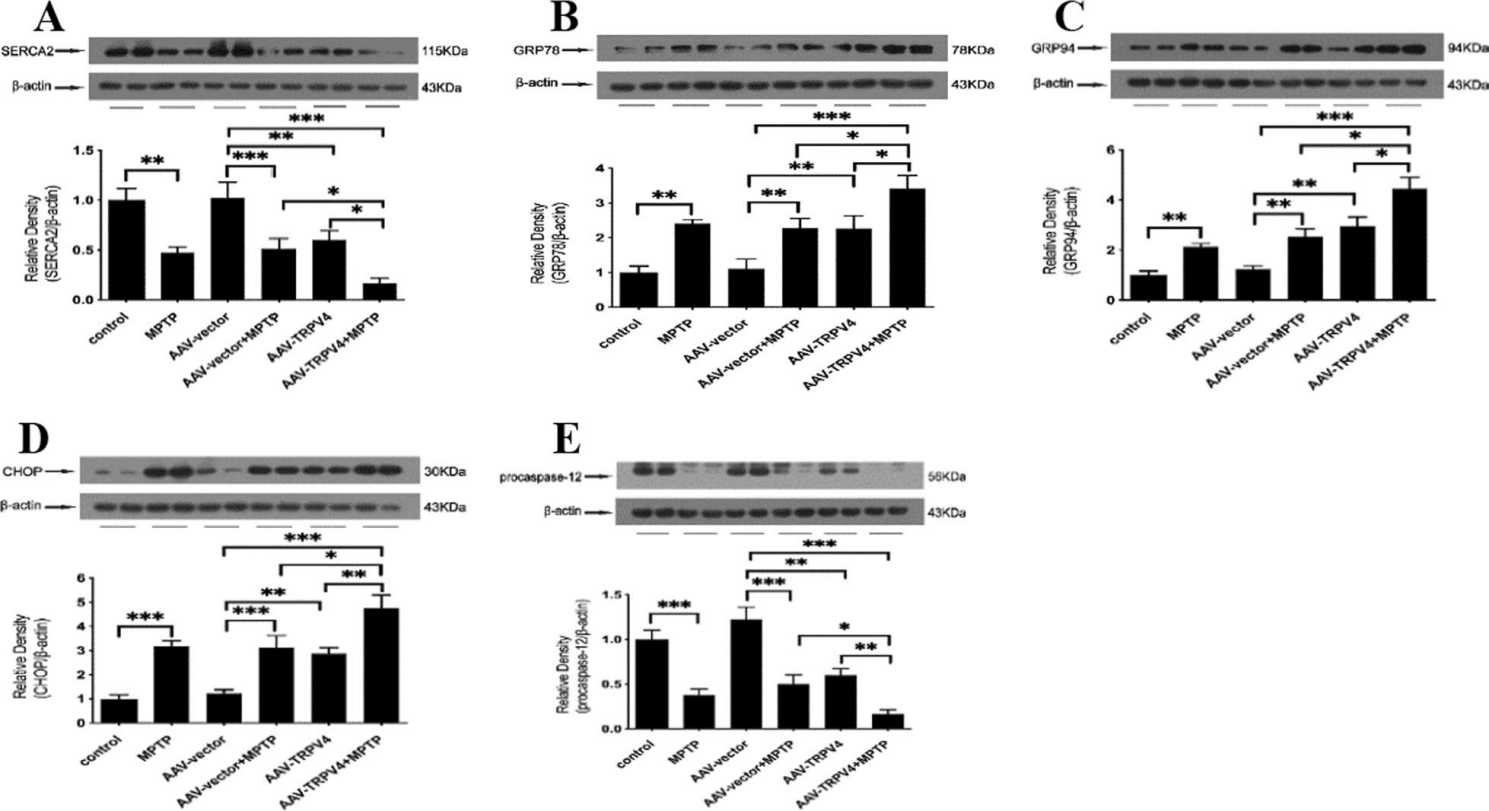
Effects of TRPV4 upregulation on MPTP-induced ER stress in the SN. After infused with 0.3 μl AAV-TRPV4 bilaterally into the SN of mice for 21 days and administrated with MPTP at 30 mg/kg/day for 7 days, the SN was rapidly dissected out. The protein levels of SERCA2, GRP78, GRP94, CHOP and procaspase-12 in the SN of mice were detected by Western blot. **A** Upregulation of TRPV4 deteriorated the MPTP-induced reduction in SERCA2 in the SN (*n* = 6). **B** Upregulation of TRPV4 exacerbated the MPTP-induced activation of GRP78 in the SN (*n* = 6). **C** Upregulation of TRPV4 exacerbated the MPTP-induced activation of GRP94 in the SN (*n* = 6). **D** Upregulation of TRPV4 exacerbated the MPTP-induced activation of CHOP in the SN (*n* = 6). **E** Upregulation of TRPV4 accelerated the MPTP-induced decrease in procaspase-12 in the SN (*n* = 6). All data are presented as mean ± SEM. Statistical significance: *P < 0.05, **P < 0.01, ***P < 0.001

To further confirm that TRPV4 has a direct effect on ER stress independent of MPTP-induced neurotoxicity, we injected AAV2/9 TRPV4 to upregulate the expression of TRPV4 in the SN. Upregulation of TRPV4 also induced ER stress. Activation of TRPV4 significantly decreased SERCA2 (Fig. 6A), activated GRP78, GRP94, CHOP and procaspase-12 (Fig. 6B–E). Moreover, we found that upregulation of TRPV4 in PD mice accelerated these changes. These data indicate that TRPV4 is an independent risk factor affecting ER homeostasis and that its effect does not rely on MPTP-induced neurotoxicity.

### Knockdown of TRPV4 in the SN attenuated inflammation induced by MPTP

**Fig. 7:**
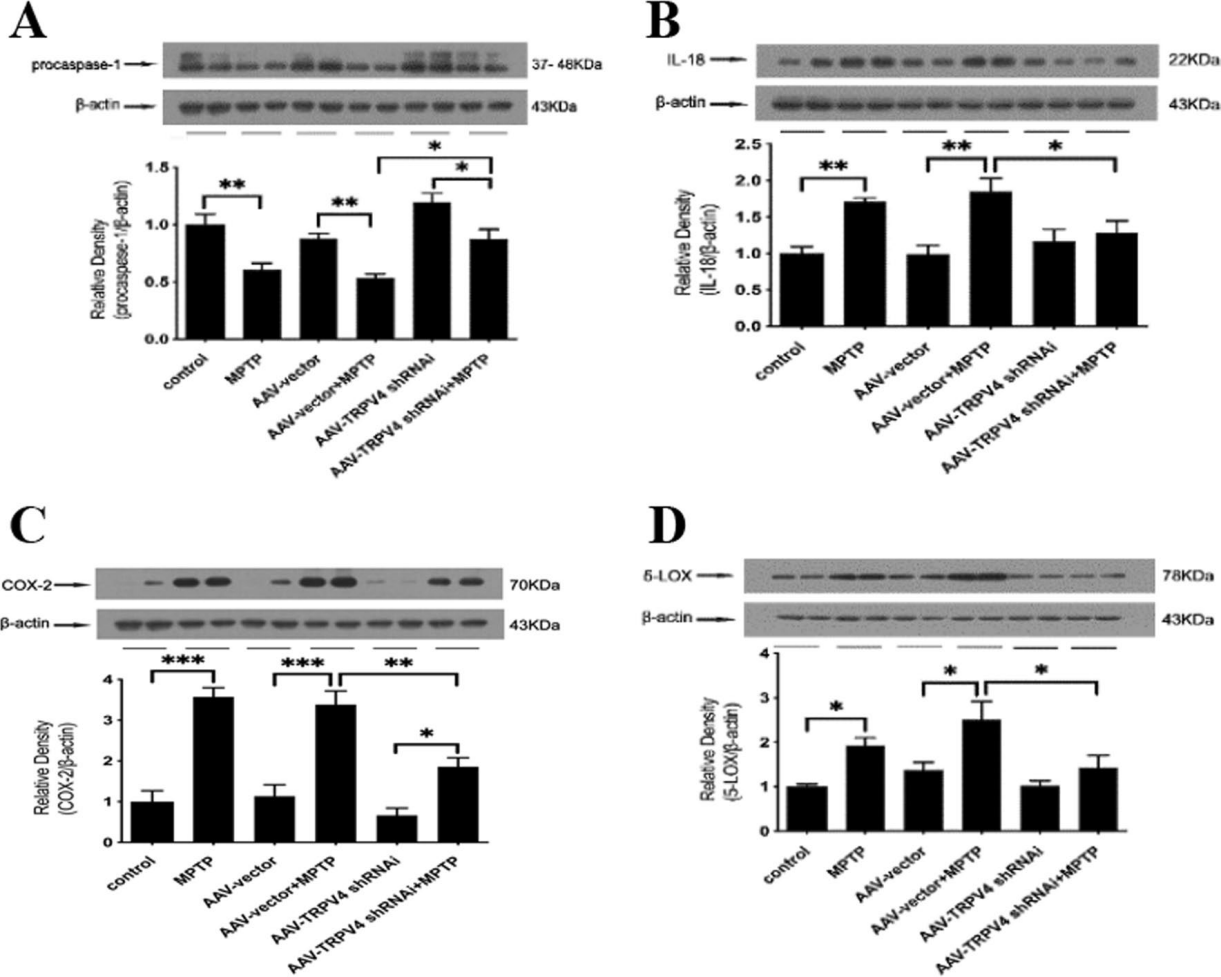
Knockdown of TRPV4 in the SN attenuated inflammation in PD mice. After infused with 0.5 μl AAV-TRPV4 shRNAi bilaterally into the SN of mice for 21 days and administrated with MPTP at 30 mg/kg/day for 7 days, the SN was rapidly dissected out. The protein levels of procaspase-1, IL-18, COX-2 and 5-LOX in the SN of mice were detected by Western blot. **A** Knockdown of TRPV4 in the SN restored the MPTP-induced the reduction in procaspase-1 (*n* = 6). **B** Knockdown of TRPV4 in the SN decreased the MPTP-induced high level of IL-18 (*n* = 6). **C** Knockdown of TRPV4 in the SN decreased the MPTP-induced high level of COX-2 (*n* = 6). **D** Knockdown of TRPV4 in the SN decreased the MPTP-induced high level of 5-LOX (*n* = 6). All data are presented as mean ± SEM. Statistical significance: *P < 0.05, **P < 0.01, ***P < 0.001

To determine whether TRPV4 plays an important role in MPTP-induced inflammation in the SN, we detected the levels of proinflammatory factors by Western blot. We found that knockdown of TRPV4 before MPTP treatment inhibited the MPTP-induced activation of procaspase-1 (Fig. 7A) and significantly reduced the high protein levels of IL-18, COX-2, and 5-LOX in the SN of PD mice (Fig. 7B–D). These results indicate that TRPV4 may mediate the inflammatory pathways and knockdown of TRPV4 has an anti-inflammatory effect.

### Upregulation of TRPV4 in the SN accelerated inflammation induced by MPTP

**Fig. 8:**
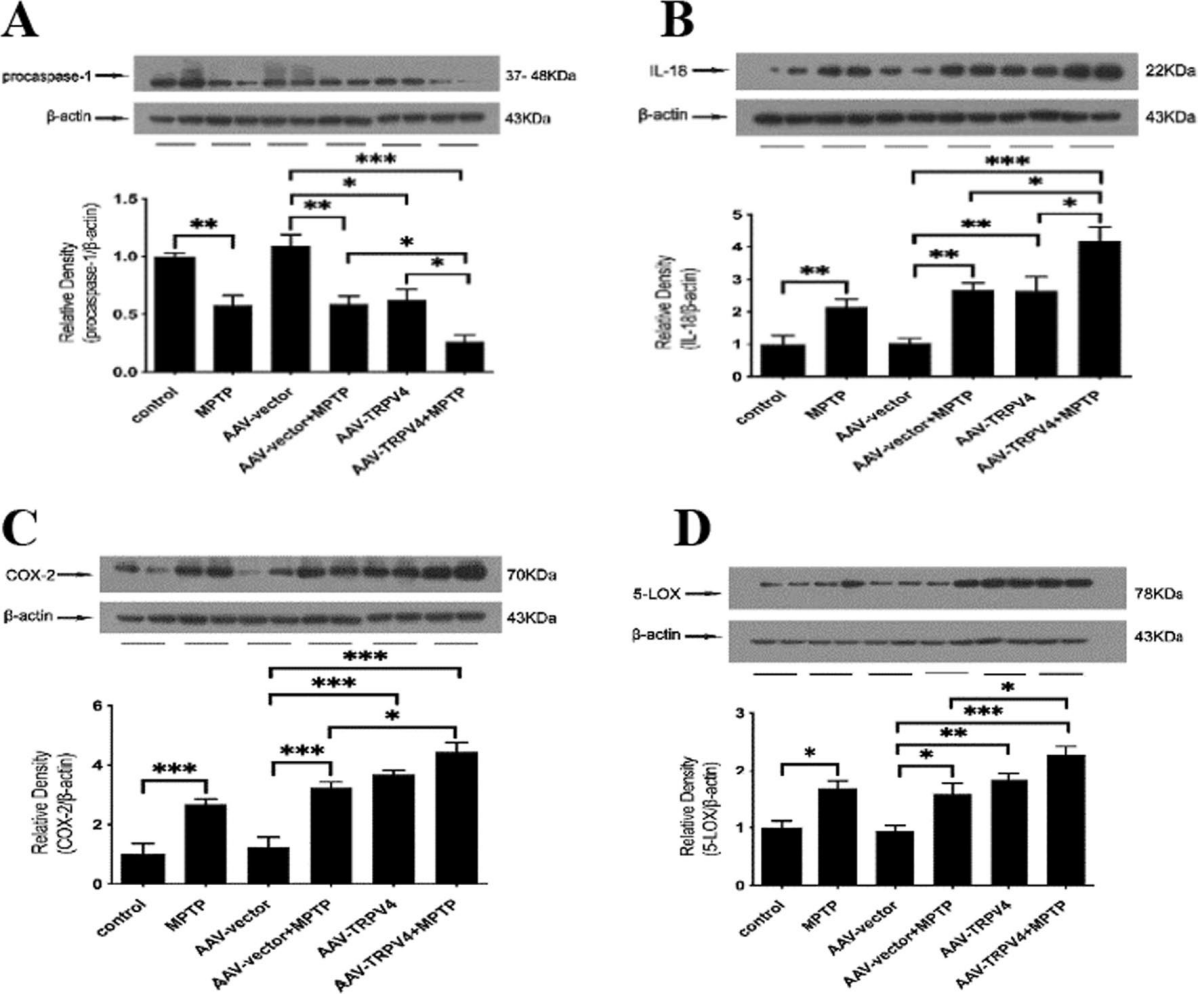
Upregulation of TRPV4 in the SN aggravated inflammation in PD mice. After infused with 0.3 μl AAV-TRPV4 bilaterally into the SN of mice for 21 days and administrated with MPTP at 30 mg/kg/day for 7 days, the SN was rapidly dissected out. The protein levels of procaspase-1, IL-18, COX-2 and 5-LOX in the SN of mice were detected by Western blot. **A** Upregulation of TRPV4 further decreased the MPTP-induced the reduction in procaspase-1 in the SN (*n* = 6). **B** Upregulation of TRPV4 further increased the MPTP-induced high level of IL-18 in the SN (*n* = 6). **C** Upregulation of TRPV4 further increased the MPTP-induced high level of COX-2 in the SN (*n* = 6). **D** Upregulation of TRPV4 further increased the MPTP-induced high level of 5-LOX in the SN (*n* = 6). All data are presented as mean ± SEM. Statistical significance: *P < 0.05, **P < 0.01, ***P < 0.001

To further confirm the role of TRPV4 in mediating the inflammatory pathways in the SN, we upregulated the expression of TRPV4 via AAV2/9 TRPV4 injection into the SN. Indeed, upregulation of TRPV4 induced inflammation. Activated TRPV4 significantly induced a reduction in procaspase-1 (Fig. 8A) and increased the levels of IL-18, COX-2, and 5-LOX (Fig. 8B–D). Moreover, we found that upregulation of TRPV4 in the SN exacerbated inflammation in PD mice. These data indicate that TRPV4 is an independent mediator of inflammation in the SN and its effect does not rely on MPTP-induced neurotoxicity.

## Discussion

TRPV4 channels are novel plasma membrane Ca^2+^ channels that are widely expressed in the brain [34]. In the current study, we first found that in the SN of MPTP-induced PD mice, the expression of TRPV4 was increased. As the normal function of Ca^2+^ channels is essential for neuronal cells, disruption of these channels might lead to several diseases, including PD [35]. To directly evaluate the contribution of TRPV4 to neurogenic damage, we used the AAV injection to mediate TRPV4 expression in the SN (Fig. 1). We found that genetic knockdown of TRPV4 alleviated locomotor deficits in MPTP-treated mice, while upregulation of TRPV4 exacerbated MPTP-induced locomotor deficits (Fig. 2). To investigate whether TRPV4 plays a role in the pathogenesis of PD mice, immunohistochemistry was performed to assess the changes in the number of TH-positive neurons in each group. TH-positive neurons are considered functional DA neurons in the SN. Mice treated with MPTP showed a decrease in the number of TH-positive neurons and Nissl-positive neurons. This phenomenon, which is the characteristic pathology of neurodegeneration in PD, is usually seen in the PD brain. Interestingly, knockdown of TRPV4 significantly increased TH-positive neuron and Nissl-positive neuron survival. Moreover, upregulation of TRPV4 further decreased the number of TH-positive neurons and Nissl-positive neurons in the SN of the PD brain (Figs. 3, 4). Altogether, these results confirmed that TRPV4 was responsible for the MPTP-induced PD phenotype.

To understand the neurotoxic role of TRPV4 in the MPTP-induced mouse model of PD, we investigated the mechanisms necessary for neuronal death. According to our previous studies in the MPP^+^-induced PC12 cell model in vitro, we measured the expression of molecules related to the ER-associated signaling pathway, which is a landmark of PD pathogenesis. Similar to ryanodine receptor (RyR) and inositol (1,4,5)-trisphosphate receptor (IP_3_R), which are Ca^2+^ release channels located in the ER, SERCA is a Ca^2+^ pump in the ER. It functions as a gatekeeper to bring Ca^2+^ into the ER to prevent Ca^2+^ homeostasis dysfunction. The major subtype SERCA2 is constitutively expressed in the brain [36]. It has been reported that TRPV4 expression upregulation by infrared radiation can trigger Ca^2+^-induced Ca^2+^ release (CICR) through the ryanodine receptor (RyR) and inositol (1,4,5)-trisphosphate receptor (IP_3_R) in neurons [37]. In the present study, we found that MPTP impaired Ca^2+^ buffering capacity by reducing SERCA2 expression, which then led to ER stress. Knockdown of TRPV4 by AAV significantly improved the function of SERCA2 and offset the cytoplasmic Ca^2+^ disturbance. The possible mechanism is that SERCA2 expression is destroyed by MPTP-induced inflammation and oxidative stress [38, 39], while TRPV4 inhibition has been identified to alleviate inflammation and oxidative stress [25, 40]. In addition, GRP78 and GRP94 are key molecular chaperones of ER stress that play crucial roles in apoptosis of DA neurons [41, 42]. It is well established that the protein levels of GRP78 and GRP94 are significantly decreased, whereas SERCA is upregulated in the dysfunctional hearts of mice [43]. This suggests that SERCA2 may be upstream of GRP78 and GRP94. In present study, GRP78 and GRP94 were markedly increased in the MPTP-treated group compared with the control group, which suggested that ER stress was induced by MPTP. Mediating TRPV4 expression in the SN by AAV significantly altered the levels of SERCA2, GRP78 and GRP94 (Figs. 5A–C and 6A–C). These results indicate that TRPV4 can mediate ER stress. To confirm our conclusions, we measured the levels of ER stress-associated apoptosis marker proteins, such as CHOP and procaspase-12 [44, 45]. Interestingly, we found that excessive ER stress was reversibly suppressed by knockdown of TRPV4 through decreasing CHOP expression and procaspase-12 activation (Fig. 5D, E). However, upregulation of TRPV4 led to the opposite outcome (Fig. 6D, E). In summary, we found that TRPV4-mediated ER stress was at least one of the mechanisms of MPTP-induced neuronal injury.

The development of effective treatments for PD partly depends on a better understanding of the regulatory mechanisms. Moreover, numerous findings from clinical studies have indicated that inflammation is one of the pathogenic mechanisms of the development of PD. It is well established that MPTP causes inflammatory responses in vivo [46, 47]. Several studies have shown the important role of TRPV4 in inflammation in different organs. It is considered a proinflammatory molecule under certain physiological conditions [47–49]. The maturation of IL-18 and caspase-1 depends on the activation of the nucleotide-binding oligomerization domain-like receptor family pyrin domain-containing 3 (NLRP3) inflammasome. Some factors, such as the elevation of intracellular free calcium concentration and lysosome disruption, are responsible for the activation of this kind of inflammasome [50]. A study proved that the TRPV4 specific inhibitor HC067047 could block the increases in NLRP3 and caspase-1 following pilocarpine-induced status epilepticus in mice, and the experimental results are consistent with our data [25]. Normally, endogenous activators of TRPV4 include heat, an acidic pH, oxidative stress and arachidonic acid (AA) [19]. AA is released by cytoplasmic phospholipase A_2_ (PLA_2_) and then converted into inflammatory eicosanoids by cyclooxygenase (COX) and lipoxygenase (LOX). Various studies indicate that TRPV4 signaling requires activation of PLA_2_ [51, 52], and Ca^2+^-dependent protein kinases activated by the influx of Ca^2+^ ([Ca^2+^]_i_) may induce COX and LOX overexpression [53–55]. Subsequently, upregulation of COX and LOX drives the production of proinflammatory factors, including prostaglandin (PG) [52] and leukotriene (LT) [56], which are linked to inflammation and inflammatory injury. This evidence demonstrates that TRPV4-depended [Ca^2+^]_i_ plays a crucial role in the AA metabolic process, which causes an inflammatory storm. In present study, we first investigated the role of TRPV4 in inflammation in the SN of PD mice. To confirm our hypothesis in this research, we measured the levels of proinflammatory molecules in the SN. We found that knockdown of TRPV4 significantly inhibited MPTP-induced activation of COX-2, 5-LOX, IL-18 and procaspase-1 (Fig. 7). In contrast, upregulation of TRPV4 significantly increased the levels of COX-2, 5-LOX, and IL-18 and decreased procaspase-1expression, which were deteriorated in PD mice (Fig. 8). These data support our hypothesis that TRPV4 acts as a mediator of neuroinflammation and may amplify the inflammatory responses.

Ca^2+^ is an important messenger responsible for cellular activities, and Ca^2+^ homeostasis dysfunction is commonly observed in neurological diseases. In present study, we found that TRPV4, a Ca^2+^-permeable channel, was involved in the MPTP-induced PD mouse model. Specifically, TRPV4 expression was upregulated in the SN by treatment with MPTP for the following reasons. First, TRPV4 was directly activated by PLA_2_ [57], the expression of which was upregulated by MPTP [58]. Second, TRPV4 was activated by mechanical stretching of the cell membrane and changes in extracellular osmolarity [11], which were elicited by MPTP-induced inflammatory edema.

**Fig. 9:**
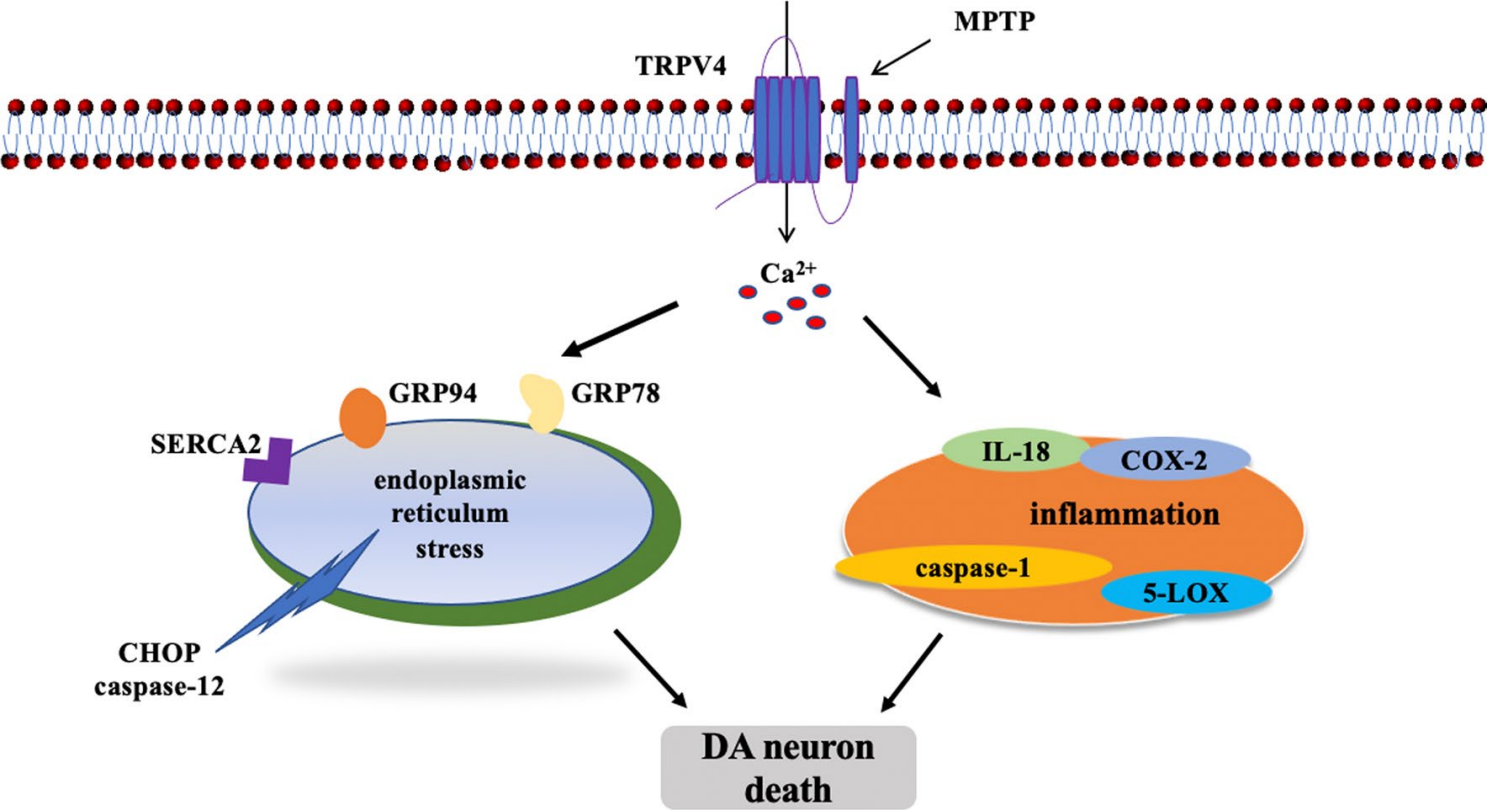
MPTP-induced activation of TRPV4 mediating ER stress and inflammation caused DA neuron death in the SN

In present study, we found that activated TRPV4 contributed to neuronal loss in the SN of PD mice via ER stress and the inflammatory pathway (Fig. 9). Notably, ER stress and neuroinflammation interact in nervous system diseases [59, 60]. It is well accepted that inflammation is activated by ER stress, and high expression of multiple proinflammatory factors is relevant to neurodegenerative diseases. Moreover, inflammation-induced ER stress is also involved in neurotoxicity. It has been proven that inhibiting ER stress attenuates inflammation-mediated neuronal dysfunction, and this effect ultimately decreases the expression of apoptosis-associated proteins in the inflammatory microenvironment [61]. Taken together, these results suggest that ER stress and inflammation are highly orchestrated processes. In present study, we found for the first time that (I) TRPV4 expression was upregulated in MPTP-induced mice. (II) TRPV4 mediated ER stress and inflammation to induce the loss of DA neurons and behavioral deficits in MPTP-induced mice, and knockdown of TRPV4 prevented these neuronal impairments. Therefore, this study may open new research avenues for the drug treatment of PD. However, the clinical application value and the potential of special chemical antagonists of TRPV4 or natural medicines and gene therapies targeting TRPV4 need to be investigated in the future.

## Conclusions

The present study demonstrated that TRPV4 regulated ER stress and inflammation to induce the loss of DA neurons and behavioral deficits of MPTP-induced mice. Knockdown of TRPV4 alleviated these neuronal impairments significantly. Therefore, targeting TRPV4 to mediate ER stress and inflammation may be a potential therapeutic strategy for PD.

## Data Availability

All data generated or analyzed during this study are included in this published article.

## Abbreviations

PD: Parkinson’s disease
TRPV4: Transient receptor potential vanilloid 4
ER: Endoplasmic reticulum
MPTP: 1-Methyl-4-phenyl-1,2,3,6-tetrahydropyridine
AAV: Adeno-associated virus
SN: Substantia nigra
TH: Tyrosine hydroxylase
DA: Dopamine
MPP^+^: 1-Methyl-4-phenylpyridinium ion
IL-6: Interleukin-6
IL-1β: Interleukin-1β
TNF-α: Tumor necrosis factor-α
TRP: Transient receptor potential
UPR: Unfolded protein response
SERCA2: Sarco/endoplasmic reticulum Ca^2^^+^-ATPase 2
GRP78: Glucose-regulated protein 78
GRP94: Glucose-regulated protein 94
procaspase-12: Pro-cysteinyl aspartate specific proteinase-12
CHOP: C/EBP homologous protein
procaspase-1: Pro-cysteinyl aspartate specific proteinase-1
COX-2: Cyclooxgenase-2
5-LOX: 5-Lipoxygenase
IL-18: Interleukin-18
CICR: Ca^2^^+^-induced Ca^2^^+^ release
RyR: Ryanodine receptor
IP_3_R: Inositol (1,4,5)-trisphosphate receptor
SERCA: Sarco/endoplasmic reticulum Ca^2^^+^-ATPase
caspase-12: Cysteinyl aspartate specific proteinase-12
NLRP3: Nucleotide-binding oligomerization domain-like receptor family pyrin domain-containing 3
AA: Arachidonic acid
PLA_2_: Phospholipase A_2_
COX: Cyclooxgenase
LOX: Lipoxygenase
